# Epigenetic alterations in hippocampus of SAMP8 senescent mice and modulation by voluntary physical exercise

**DOI:** 10.3389/fnagi.2014.00051

**Published:** 2014-03-20

**Authors:** Marta Cosín-Tomás, María J. Alvarez-López, Sandra Sanchez-Roige, Jaume F. Lalanza, Sergi Bayod, Coral Sanfeliu, Merce Pallàs, Rosa M. Escorihuela, Perla Kaliman

**Affiliations:** ^1^Unitat de Farmacologia, Facultat de Farmàcia Institut de Biomedicina Universitat de Barcelona (IBUB), Nucli Universitari de PedralbesBarcelona, Spain; ^2^Department of Brain Ischemia and Neurodegeneration, Institut d'Investigacions Biomèdiques de Barcelona (IIBB)-Consejo Superior de Investigaciones Científicas (CSIC)Barcelona, Spain; ^3^Departamento de Psiquiatría y Medicina Legal, Facultad de Medicina, Instituto de Neurociencias, Universitat Autònoma de BarcelonaBarcelona, Spain

**Keywords:** exercise, aging, Alzheimer, SAMP8, microRNAs

## Abstract

The senescence-accelerated SAMP8 mouse model displays features of cognitive decline and Alzheimer's disease. With the purpose of identifying potential epigenetic markers involved in aging and neurodegeneration, here we analyzed the expression of 84 mature miRNAs, the expression of histone-acetylation regulatory genes and the global histone acetylation in the hippocampus of 8-month-old SAMP8 mice, using SAMR1 mice as control. We also examined the modulation of these parameters by 8 weeks of voluntary exercise. Twenty-one miRNAs were differentially expressed between sedentary SAMP8 and SAMR1 mice and seven miRNAs were responsive to exercise in both strains. SAMP8 mice showed alterations in genes involved in protein acetylation homeostasis such as *Sirt1* and *Hdac6* and modulation of *Hdac3* and *Hdac5* gene expression by exercise. Global histone H3 acetylation levels were reduced in SAMP8 compared with SAMR1 mice and reached control levels in response to exercise. In sum, data presented here provide new candidate epigenetic markers for aging and neurodegeneration and suggest that exercise training may prevent or delay some epigenetic alterations associated with accelerated aging.

## Introduction

Epigenetic changes are currently recognized as part of the aging process and have been implicated in many age-related chronic diseases (Jakovcevski and Akbarian, 2012; Akbarian et al., 2013; Lopez-Otin et al., 2013). The term epigenetics includes a variety of processes known to regulate gene expression in a stable and potentially reversible way, without altering the primary DNA sequence (Jaenisch and Bird, 2003). The molecular mechanisms that mediate epigenetic regulation are principally DNA methylation, post-translational modifications of the histones, and regulation by non-coding RNAs. Among the latter, microRNAs (miRNAs) are small molecules (22 nucleotides approximately) that regulate gene expression by binding to its target messenger RNA (mRNA) inhibiting its translation, or, less frequently, promoting its degradation (Bartel, 2009). To date, around 700–800 miRNAs have been identified in the human genome (Bentwich et al., 2005). Altered expression of miRNAs has been described in different chronic pathologies and they are currently considered to be critical in the aging process (Jung and Suh, 2012). miRNAs seem to play an important role in the developing nervous system, in the physiology of high-order brain functions such as learning, memory, and emotion regulation, and in the manifestation of neurological disorders such as amyotrophic lateral sclerosis, Tourette's syndrome, Alzheimer's disease (AD) and others (Yang et al., 2007; Mastroeni et al., 2011; Goldie and Cairns, 2012; Van Den Hove et al., 2014). On the other hand, histone covalent modifications have also been implicated in the aging process (Dang et al., 2009; Greer et al., 2010; Siebold et al., 2010; Di Bernardo et al., 2012; Huidobro et al., 2013; Tammen et al., 2013). Histone acetyltransferases (HATs) and histone deacetylases (HDACs) are among the best characterized histone modifying enzymes in neurons (Crepaldi and Riccio, 2009). HATs transfer an acetyl group to the amino groups of histone lysine residues and generally increase DNA transcription. For their part, HDACs decrease DNA accessibility by deacetylation of histone lysines (Legube and Trouche, 2003). An adequate balance between HAT and HDAC levels and activity is crucial for neuronal homeostasis and for brain functions such as learning and memory (Saha and Pahan, 2006). Notably, alterations in histone acetylation levels have been observed in several models of neurodegenerative diseases, including AD (Scheff et al., 2007; Arendt, 2009).

It has been widely reported that the regular practice of physical exercise improves brain health and provides cognitive and psychological benefits (Kaliman et al., 2011). Some of the neurophysiological effects of physical exercise have been attributed to changes in the transcriptional profiles of growth and neurotrophic factors such as IGF1 and BDNF (Dishman et al., 2006; Gomez-Pinilla et al., 2008; Trejo et al., 2008; Alvarez-Lopez et al., 2013). Recent data have described the positive impact of physical exercise on epigenetic alterations in the rodent brain (Chandramohan et al., 2008; Collins et al., 2009; Abel and Rissman, 2013; Lovatel et al., 2013).

The spontaneous senescence-accelerated P8 mouse model (SAMP8) is currently considered a model of AD (Pallas et al., 2008a; Morley et al., 2012b; Cheng et al., 2013a,b; Wang et al., 2013). Indeed, SAMP8 mice display cognitive and behavioral alterations which are accompanied by molecular features typical of AD such as overproduction of amyloid-beta protein, increased tau phosphorylation, cholinergic deficits in the forebrain and increased oxidative stress (Takeda, 2009; Del Valle et al., 2011; Morley et al., 2012a). SAMP8 mice were phenotypically selected from AKR/J, and SAM resistant mice (SAMR1), which have a similar genetic background, have been extensively used as a control model because they show normal aging characteristics (Takeda et al., 1991). With the purpose of identifying potential epigenetic markers involved in aging and neurodegeneration, here we studied the expression levels of a set of 84 mature miRNAs with reported effects on neurological development and disease, the expression of several genes involved in maintenance of the histone acetylation balance (HATs and HDACs) and the levels of histone global acetylation (H3ac, H4ac) in the hippocampus of 8-month-old SAMP8 and SAMR1 mice. We also explored the impact of 8 weeks of voluntary wheel running intervention on these parameters. Through these analyses, we identified hippocampal epigenetic factors that are altered in the senescent SAMP8 mice, some of which were modulated by physical exercise.

## Materials and methods

### Animal care and voluntary exercise paradigm

All experimental procedures were approved by the Ethics Committee of the University of Barcelona (Comissió Ètica d'Experimentació Animal UB), following the “Principles of laboratory animal care” and were carried out in accordance to the European Communities Council Directive (86/609/EEC).

SAMP8 and SAMR1 female mice were provided by El Parc Tecnològic (Barcelona, Spain) and were maintained under standard conditions (temperature 23 ± 1°C, humidity 50–60%, 12:12-h light-dark cycle, lights on at 7:00 a.m.), with food (A04, Harlan, Spain) and tap water available *ad libitum* throughout the study. Body weight (g) was measured weekly. This study was performed in female mice as sex differences in the patterns of voluntary exercise in mice have been reported (Alvarez-Lopez et al., 2013).

The running wheels (ENV-044 Mouse Low-Profile Wireless Running Wheel, Med Associates Inc.; 15.5 cm circumference; 25° from horizontal plane) were located in the animal colony room inside cages 19 cm high × 27 cm wide × 40 cm deep. Wheel-running activity was monitored through a wireless transmitter system by using a Hub located in the same animal colony room. Wireless Running Wheel Manager Data Acquisition Software (SOF-860; Med Associates Inc.) recorded the activity and time of each wheel revolution, which occurred whenever a magnet attached to the wheel's axis made contact with an electronic switch sending a signal to the Hub. Although revolutions were monitored continuously, voluntary activity occurred primarily during the dark phase. The running mice were placed individually in the large cages and had unlimited access to a running wheel 7 days a week for 8 weeks. Control mice were placed individually in cages of equal size without a running wheel.

At the end of the intervention all mice were 8-month-old. Animals were sacrificed by decapitation and the brains were dissected on ice to obtain the hippocampus. Tissues were immediately frozen and stored at −80°C for further analysis.

### Plasma analysis

Blood samples were collected in 5% EDTA-tubes at the time of death for IGF1, cholesterol, and triglycerides determination. Plasma was obtained by centrifugation (3500 rpm, 10 min, room temperature) and stored at −80°C.

IGF1 was determined by the ELISA kit Mouse/Rat Insulin-like Growth Factor-I; (Mediagnost, IGF-I EIA E25, Reutlingen, Germany) following the manufacturer's recommendations.

Plasma triglyceride and cholesterol concentrations were measured by using the colorimetric tests (Triglyceride L-type and Cholesterol kit, respectively), from Wako Chemicals GmbH (Neuss, Germany).

### Total RNA extraction

Total RNA was extracted using *mir*Vana™ RNA Isolation Kit (Applied Biosystems) according to the instructions of the manufacturer. The yield, purity and quality of RNA were determined spectrophotometrically (NanoDrop, USA) and using the Bioanalyzer 2100 capillary electrophoresis. RNAs with 260/280 ratios and RIN higher than 1.9 and 7.5, respectively, were selected.

### Real-time quantitative PCR

Random-primed cDNA synthesis was performed at 37°C starting with 0.3 μ g of RNA, using the High Capacity cDNA Archive kit (Applied Biosystems). Gene expression was measured in an ABI Prism 7900HT Real Time PCR system using TaqMan FAM-labeled specific probes (Applied Biosystems). A list of the probes used is presented in Supplementary Table 1 (S1). Results were normalized to *TATA-binding protein (Tbp)* expression.

### Western blots

Histone fractions (5 μ g) were electrophoretically analyzed on 12% bis-Tris polyacrylamide gels and transferred to a 0.45 μm PVDF membrane. Membranes were blocked for 1 h with 5% BSA in PBS and incubated overnight at 4°C with the specific primary antibodies (1:1000, Millipore). Membranes were washed and incubated with peroxidase-labeled secondary antibodies at room temperature for 1 h. Immunoreactive bands were detected by autoradiography. Specific bands from Western blot were quantified by scanning densitometry using Quantity One® 1-D analysis 4.6.3. software (Bio-Rad USA, Life Science Research, Hercules, CA). Histone modifications levels were corrected by total histone expression.

### microRNA expression array

RNA samples from 16 female individuals (four from each group: sedentary SAMR1, runner SAMR1, sedentary SAMP8, runner SAMP8) were converted to cDNA through a reverse transcription reaction using miScript II RT Kit (Qiagen, Hilden Germany) according to the manufacturer's instructions. The expression of 84 mature miRNAs was then analyzed using the *miScript® miRNA PCR Array-Neurological Development and Disease miRNA PCR Array* (Qiagen). miRNAs expression was measured in an ABI Prism 7900HT through SYBR-green-based real time PCR. The data obtained were processed with the online software “*Web-based miScript miRNA PCR Array data analysis tool.”* The mean of the relative gene expression of the small non-coding RNAs SNORD61, SNORD68, SNORD72, SNORD95, SNORD96A was used to normalize results since they presented similar expression levels across the individuals and groups and the lowest standard deviations among all the housekeeping miRNAs proposed.

### Statistical analysis

The statistical analysis was performed using the Statistical Package for Social Sciences (SPSS, version 19.0). The Two-Way ANOVA analysis of variance [2 strains (R1,P8) × 2 conditions (sedentary, runner)] was conducted to assess strain and exercise intervention effects. Comparisons between groups were performed by two-tailed Student's *t*-test for independent samples; *p*-values below 0.05 were considered statistically significant. Statistical outliers (≥two standard deviations from the mean) were removed from the analyses. Functional prediction analysis (Supplementary information S3–S6) was only performed for those miRNAs significantly altered with a *p* < 0.05 and a magnitude of effect ≥1.4.

## Results

### Positive effects of 8 weeks of voluntary wheel running in hippocampal gene expression and IGF1 plasma levels

**Figure 1:**
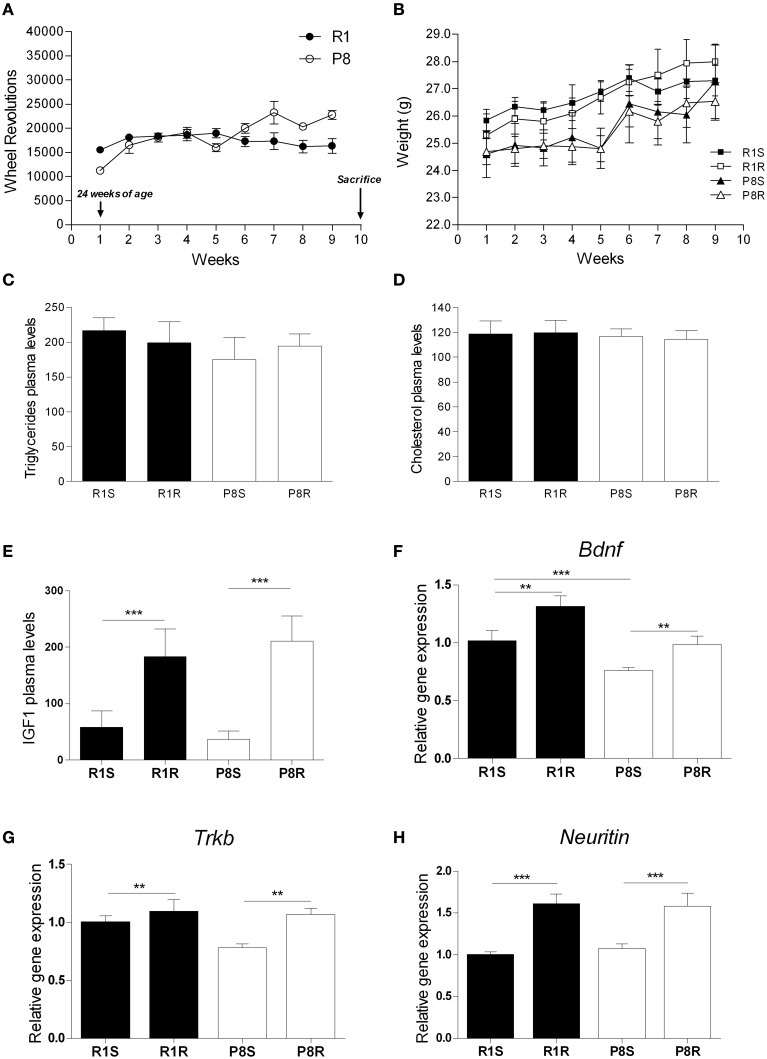
**Beneficial effects of 8 weeks of exercise training in SAMP8 senescent mice**. (**A**) Mean wheel revolutions/week register in exercised SAMR1 and SAMP8 mice; **(B)** Body weight (g) mean during exercise intervention in sedentary and exercised groups from both SAMR1 and SAMP8 strains. **(C)** Triglyceride plasma levels (mg/dL). **(D)** Cholesterol plasma levels (mg/dL). **(E)** IGF1 plasma levels (ng/mL). **(F–H)** Gene expression of *Bdnf* **(F)**, *Trkb* **(G)**, and *Neuritin* **(H)**. Gene expression was measured by real-time PCR analysis from hippocampal mRNA using TaqMan FAM-labeled specific probes and expressed relative to *TBP* (*n* = 3–4/group for IGF1 and *n* = 5–8/group for all other measures). Mean ± standard error are represented; Two-Way ANOVA results are indicated as ^**^p < 0.01; ^***^p < 0.001.

We analyzed the effects of 8 weeks of voluntary wheel running in 6-month-old SAMP8 and SAMR1 mice. Both strains displayed a similar and stable number of wheel revolutions/week over time throughout the intervention (SAMR1, 17762 ± 2221.9 average wheel revolutions/week; SAMP8, 18018 ± 3035.7 average wheel revolutions/week, Figure 1A). No changes were found in body weight, plasma triglycerides, or plasma cholesterol between the experimental groups (Figures 1B–D; Table 1).

**Table 1:** **Two-Way ANOVA analysis was used to compare plasma cholesterol, triglycerides and IGF1 levels and hippocampal expression of neurotrophic genes in sedentary and exercised 8-month-old SAMR1 and SAMP8 mice**.

	**Two-way ANOVA analysis**								
	**Exercise**			**Strain**			Strain *exercise		
	***df***.	***F***	***p-value***	***df***.	***F***	***p-value***	***df***.	***F***	***p-value***
**PLASMA**									
IGF1	1, 10	19.716	**<0.001**	1, 10	0.008	0.932	1, 10	0.528	0.484
Cholesterol	1, 21	0.045	0.835	1, 21	<0.001	0.998	1, 21	0.147	0.705
Triglycerids	1, 21	0.913	0.35	1, 21	0.007	0.933	1, 21	0.794	0.383
**HIPPOCAMPAL GENES**									
*Bdnf*	1, 22	12.16	**0.002**	1, 22	15.285	**<0.001**	1, 22	0.233	0.634
*TrkB*	1, 21	8.239	**0.009**	1, 21	3.729	**0.067**	1, 21	2.26	0.148
*Neuritin*	1, 23	31.931	**<0.001**	1, 23	0.048	0.828	1, 23	0.83	0.372

*Natural log, square root, and inverse transformation were applied to normalize plasma cholesterol, plasma triglycerides, and Neuritin distribution, respectively. P-values < 0.05 were considered statistically significant. df stands for degrees of freedom. Bold values correspond to statistically significant p-values*.

To confirm the efficacy of the exercise intervention, we analyzed several factors previously reported to be modulated in rodents in response to physical training (e.g., plasma IGF1 and hippocampal *Bdnf* gene expression) (Kaliman et al., 2011). Both exercised SAMR1 and SAMP8 mice showed significantly increased IGF1 plasma levels compared with their corresponding sedentary groups (Figure 1E; Table 1).

In the hippocampus, *Bdnf* gene was underexpressed in sedentary SAMP8 compared with SAMR1 mice and both *Bdnf* and its receptor *TrkB* were significantly upregulated in response to the exercise intervention in both strains (Figures 1F,G; Table 1). Notably, after the exercise intervention *Bdnf* levels in SAMP8 mice were undistinguishable from those found in sedentary SAMR1 controls [*t*_(12)_ = 0.279, *p* = 0.785] (Figure 1F). *Neuritin* gene, a well characterized target of BDNF, was upregulated in both strains by exercise training (Figure 1H; Table 1).

These results confirmed that the wheel running intervention was effective and therefore represents a good model to explore epigenetic effects of exercise in the SAMP8 mice.

### microRNA expression profile is altered in SAMP8 hippocampus and modulated by physical exercise

We compared the microRNA expression profiles between 8-month-old SAMP8 and SAMR1 mice in order to identify putative senescence markers in the hippocampus. We used a miRNA PCR array which analyzes 84 different mouse miRNAs known to be altered in neurological diseases or involved in neuronal development. We found 18 miRNAs altered in sedentary SAMP8 compared with SAMR1 mice which were unresponsive to exercise, three miRNAs altered in SAMP8 and modulated by exercise and four miRNAs that were similarly expressed in SAMP8 and SAMR1 mice and modulated by exercise in both strains. Two-Way ANOVA analysis of this set of miRNAs are shown in Table 2. Statistical analysis of miRNAs similarly expressed in SAMP8 and SAMR1 mice and unresponsive to the exercise intervention are shown in Supplementary Table 2 (S2).

**Table 2:** **miRNAs significantly altered in SAMP8 mice and/or modulated by exercise**.

	**miRNAs**	**Two-way ANOVA analysis**								
		**Exercise**			**Strain**			Strain *exercise		
		***df.***	***F***	***p-value***	***df.***	***F***	***p-value***	***df.***	***F***	***p-value***
Strain effect; no exercise effect	let_7i_5p	1, 12	0.029	0.867	1, 12	24.140	**<0.001**	1, 12	1.252	0.285
	miR_29a_3p	1, 12	1.933	0.190	1, 12	25.847	**<0.001**	1, 12	0.609	0.450
	miR_29c_3p	1, 12	0.347	0.567	1, 12	14.705	**0.002**	1, 12	0.908	0.359
	miR_30a_5p	1, 12	0.240	0.633	1, 12	16.725	**0.001**	1, 12	0.062	0.807
	miR_30e_5p	1, 12	0.623	0.445	1, 12	8.079	**0.015**	1, 12	0.717	0.414
	miR_125b_5p	1, 12	0.236	0.636	1, 12	21.299	**0.001**	1, 12	0.195	0.666
	miR_128_3p	1, 12	0.750	0.404	1, 12	13.127	**0.003**	1, 12	0.454	0.513
	miR-138-5p	1, 12	0.169	0.688	1, 12	18.876	**0.001**	1, 12	3.598	0.082
	miR_139_5p	1, 12	2.264	0.158	1, 12	14.273	**0.003**	1, 12	1.019	0.333
	miR_140_5p	1, 12	0.643	0.438	1, 12	42.614	**<0.001**	1, 12	1.355	0.267
	miR_146b_5p	1, 12	0.051	0.825	1, 12	8.750	**0.012**	1, 12	0.152	0.704
	miR_181a_1_3p	1, 11	0.300	0.595	1, 11	9.290	**0.011**	1, 11	0.001	0.978
	miR_181a_5p	1, 12	0.480	0.502	1, 12	8.303	**0.014**	1, 12	1.962	0.187
	miR_194_5p	1, 11	0.667	0.431	1, 11	16.876	**0.002**	1, 11	0.029	0.869
	miR_337_3p	1, 12	2.061	0.177	1, 12	5.767	**0.033**	1, 12	0.160	0.696
	miR_342_3p	1, 12	0.749	0.404	1, 12	5.188	**0.042**	1, 12	1.318	0.273
	miR_431_5p	1, 12	0.330	0.576	1, 12	6.052	**0.03**	1, 12	0.479	0.502
	miR_455_5p	1, 12	4.264	0.061	1, 12	6.990	**0.021**	1, 12	0.018	0.896
Strain and exercise effect	miR-28a-5p	1, 12	7.241	**0.020**	1, 12	9.073	**0.011**	1, 12	0.435	0.522
	miR-98-5p	1, 12	12.167	**0.004**	1, 12	22.598	**<0.001**	1, 12	2.548	0.136
	miR-148b-3p	1, 12	12.421	**0.004**	1, 12	69.076	**<0.001**	1, 12	0.027	0.872
No strain effect; exercise effect	miR-7a-5p	1, 12	8.513	**0.013**	1, 12	0.200	0.663	1, 12	0.805	0.387
	miR-15b-5p	1, 12	6.094	**0.030**	1, 12	0.279	0.607	1, 12	0.546	0.474
	miR-105	1, 11	9.680	**0.010**	1, 11	1.004	0.338	1, 11	0.003	0.954
	miR-133b-3p	1, 12	7.830	**0.016**	1, 12	2.739	0.124	1, 12	0.823	0.382

*Two-Way ANOVA analysis was used to compare the hippocampal miRNAs gene expression in sedentary and exercised 8-month-old SAMR1 and SAMP8 mice. P-values < 0.05 were considered statistically significant. df stands for degrees of freedom. Bold values correspond to statistically significant p-values*.

Among the miRNAs that were significantly upregulated in SAMP8 compared with SAMR1 mice, miR-30e-5p, miR-125b-5p, and miR-128-3p have also been reported to be upregulated in post-mortem human AD hippocampus (Lukiw, 2007; Cogswell et al., 2008). Similarly, we found an increased expression of let-7i-5p, miR-29a-3p, miR-29c-3p, miR-30a-5p, miR-98-5p, miR-138-5p, miR-139-5p, miR-140-5p, miR-146b-5p, miR-148b-3p, miR-181a-1-3p, miR-181a-5p, miR-194-5p, and miR-342-3p, all of which have been reported to be altered in different AD tissues (Cogswell et al., 2008; Hebert et al., 2008; Maes et al., 2009; Wang et al., 2011, 2012; Lau et al., 2013). The rest of the differentially expressed miRNAs between strains have been found to be altered in different neurodegenerative models (28a-5p, miR-337-3p, miR-431-5p, miR-455-5p). The functional information available in the literature for these miRNAs in the central nervous system (CNS) is summarized in Supplementary Table 3 (S3).

**Figure 2:**
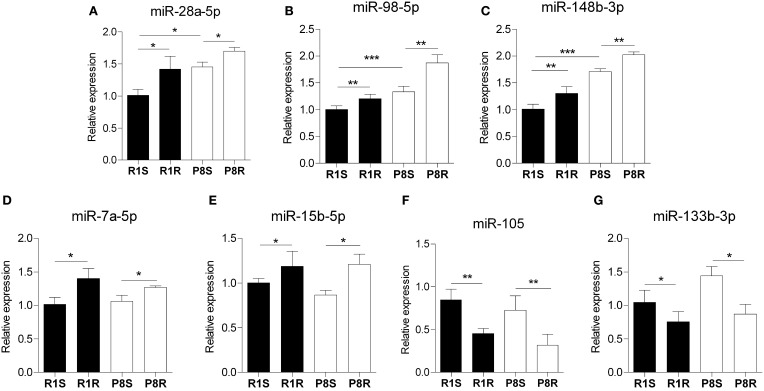
**Hippocampal microRNAs modulated by exercise in SAMR1 and SAMP8 mice**. MicroRNA expression was measured using *miScript*® miRNA PCR Array- Neurological Development and Disease miRNA PCR Array (Qiagen) and expressed relative to housekeeping miRNAs proposed by the array. **(A–C)** miRNAs that are altered in sedentary SAMP8 compared with sedentary SAMR1 mice and regulated by exercise in senescent mice, (miR-28a-5p, miR-98-5p, and mir-148b-3p, respectively). **(D–G)** miRNAs unaltered in sedentary SAMP8 mice but responsive to exercise intervention in SAMR1 and SAMP8 mice (miR-7a-5p, miR-15b-5p, miR-105, and miR-133b-3p, respectively). Means ± standard error are represented; Two-Way ANOVA results are indicated as ^*^p < 0.05; ^**^p < 0.01; ^***^p < 0.001; *n* = 4/group.

Interestingly, miR28a-5p, miR-98-5p, and miR-148b-3p expression was significantly higher in sedentary SAMP8 compared with sedentary SAMR1 mice and this difference was further accentuated by exercise (Figures 2A–C). In addition, we found that miR-7a-5p, miR-15b-5p, miR-105, and miR-133-3p exhibited similar expression levels in sedentary strains but were similarly modulated by exercise in SAMP8 and SAMR1 mice (Figures 2D–G). Functional information available in the literature for the role in the CNS of the miRNAs regulated by exercise is summarized in Supplementary Table 4 (S4).

### Alterations in the expression of HAT and HDAC genes and in global histone modifications in hippocampus of sedentary SAMP8: effects of exercise

Alterations in histone acetylation levels have been observed in several models of neurodegenerative diseases. Therefore, we analyzed in sedentary and exercised SAMP8 and SAMR1 mice, the hippocampal expression of the HAT *P300* and the NAD+ dependent HDAC *Sirt1*, both of which have been implicated in AD pathogenesis (Pallas et al., 2008b; Min et al., 2010) as well as a group of NAD+ independent HDACs (*Hdac1, Hdac2, Hdac3, Hdac5, Hdac6*).

**Figure 3:**
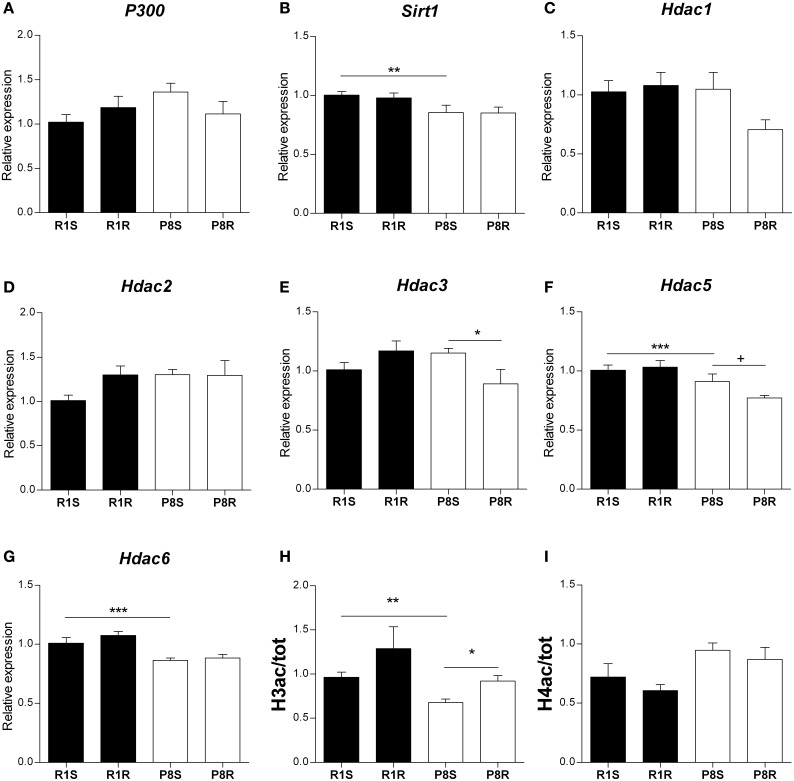
**Histone acetylation regulatory genes and global histone modifications in hippocampus of sedentary and exercised 8 months-old SAMR1 and SAMP8 mice**. Gene expression was measured by real-time PCR analysis from hippocampal mRNA using TaqMan FAM-labeled specific probes and expressed relative to *TBP* (*n* = 5–8/group). **(A)** Histone acetyltransferase *P-300*. **(B)** *Sirtuin1*. **(C–G)** Histone deacetylase *1–6, respectively*. Means ± standard error are represented; Two-Way ANOVA results are indicated as ^*^p < 0.05; ^**^p < 0.01; ^***^p < 0.001. **(H,I)** Global acetylation levels of histone H3 **(H)** and histone H4 **(I)** in hippocampus from sedentary and exercised SAMR1 and SAMP8 mice. Specific bands from Western blot were quantified by scanning densitometry (*n* = 4/group). Histone modifications were corrected by total histone and results were analyzed by two-tailed Student's *t*-test for independent samples. Means ± standard error are represented (^+^*p* < 0.1; ^*^p < 0.05; ^**^p < 0.01; ^***^p < 0.001).

We did not find any differences between strains or any modulation with exercise in histone acetyltransferase *P300* gene expression (Table 3, Figure 3A). We found lower expression levels of the histone deacetylases *Sirt1*, *Hdac5*, and *Hdac6* in sedentary SAMP8 compared with SAMR1 mice (Table 3, Figures 3B,F,G) while no significant differences between strains were detected for *Hdac1, Hdac2 and Hdac3* (Table 3, Figures 3C–E).

**Table 3:** **Two-Way ANOVA was used to compare the hippocampal expression of histone acetylation regulatory genes in sedentary and exercised 8-month-old SAMR1 and SAMP8 mice**.

**Hippocampal genes**	**Two-way ANOVA analysis**								
	**Exercise**			**Strain**			Strain *exercise		
	***df.***	***F***	***p-value***	***df.***	***F***	***p-value***	***df.***	***F***	***p-value***
*P300*	1, 23	0.106	0.748	1, 23	1.261	0.273	1, 23	2.942	0.1
*Sirt1*	1, 23	0.091	0.765	1, 23	8.369	**0.008**	1, 23	0.041	0.841
*Hdac1*	1, 23	1.128	0.299	1, 23	2.055	0.165	1, 23	1.26	0.273
*Hdac2*	1, 23	2.66	0.117	1, 23	0.221	0.643	1, 23	2.823	0.106
*Hdac3*	1, 23	0.054	0.818	1, 23	0.383	0.542	1, 23	5.249	**0.031**
*Hdac5*	1, 23	1.414	0.246	1, 23	14.639	**<0.001**	1, 23	3.104	**0.091**
*Hdac6*	1, 23	1.482	0.236	1, 23	21.536	**<0.001**	1, 23	0.453	0.508

*Quadratic transformation was applied to normalize Hdac3 distribution. P-values < 0.05 were considered statistically significant. df stands for degrees of freedom. Bold values correspond to statistically significant p-values*.

Voluntary exercise led to a significant decrease in *Hdac3* gene expression exclusively in SAMP8 mice (Table 3, Figure 3E). ANOVA analysis showed a downregulation tendency for *Hdac5* gene in exercised compared with sedentary SAMP8 mice, and this effect was found to be statistically significant by two-tailed Student's *t*-test for independent samples [P8R vs. P8S, Hdac3: *t*_(9)_ = 1.931, *p* = 0.084; Hdac5: *t*_(12)_ = 2.27, *p* = 0.042] (Table 3, Figure 3F). We did not detect any influence of the exercise intervention on *Sirt1*, *Hdac1*, *Hdac2*, and *Hdac6* gene expression (Table 3, Figures 3B–D,G).

Finally, we found that the global acetylation levels of histone H3 (H3ac) were lower in sedentary SAMP8 than in SAMR1 mice [P8S vs. R1S, *t*_(6)_ = 3.929, *p* = 0.008] and significantly increased upon exercise only in the senescent mice [P8R vs. P8S, *t*_(6)_ = −3.399, *p* = 0.019] (Figure 3H). In contrast, the acetylation of histone 4 (H4ac) did not show significant differences between groups (Figure 3I).

## Discussion

Here we explored the epigenetic alterations in the hippocampus of SAMP8 female mouse and the modulatory effect of voluntary physical exercise on the expression of several miRNAS, histone deacetylase genes and in the global acetylation level of histone H3.

Our data and those of others (Liang et al., 2009) suggest that miRNAs are involved in the down-regulation of target genes that control accelerated senescence. Indeed, we found a general upregulation pattern of miRNAs in the hippocampus SAMP8 compared with SAMR1 mice. Most of these miRNAs have also been found dysregulated in different tissues from AD patients. Notably, our study highlights the upregulation of miR-30e-5p, miR-125b-5p, and miR-128-3p as common epigenetic features in the hippocampus of SAMP8 mice and post-mortem hippocampus from AD patients. Moreover, our results support bioinformatic data by Cheng et al. who have recently predicted from a whole genome microarray study that miR-125b-5p may be involved in the brain aging phenotype of SAMP8 mice (Cheng et al., 2013a). Therefore, these miRNAs emerge as potential AD biomarkers and our data provide further support for the suitability of the SAMP8 model for future studies to explore their role on the onset and progression of AD. Supplementary Table 3 (S3) summarizes the available literature regarding the brain distribution and function of the miRNAs that we found altered in SAMP8 compared with SAMR1 sedentary mice. Bioinformatic pathway analysis (DIANA-miRPath v.2.1) indicates that these miRNAs are involved in neural processes such as neurotransmitters synapses (acetylcholine, glutamate, dopamine), long-term potentiation, axon guidance and neurotrophin signaling (S5).

Exercise training led to the increase of IGF1 in plasma and the upregulation of BDNF and other neurogenic factors in hippocampus of SAMP8 and SAMR1 strains (Figure 1). These data confirm that the intervention used in our study was effective as such effects have previously been reported in a variety of rodent models in response to exercise (Saltiel and Kahn, 2001; Llorens-Martin et al., 2010; Chang et al., 2011; Kaliman et al., 2011; Sakurai et al., 2011; Higashi et al., 2012; Alvarez-Lopez et al., 2013). We found that miR-28a-5p, miR-98a-5p, miR-148b-3p were altered in sedentary SAMP8 compared with SAMR1 mice and changed their expression levels in response to exercise (putative aging markers responsive to exercise). On the other hand, miR-7a-5p, miR-15b-5p, miR-105, miR-133b-3p, which were similarly expressed in SAMP8 and SAMR1 mice, were modulated by exercise in both strains (putative markers of exercise unrelated to aging). The data available on the function and expression of these miRNAs in the CNS are summarized in Supplementary Table 4 (S4). Further study is warranted to explore the precise mechanistic links between these miRNAs and the protective central effects of physical exercise. In this context, a prediction through bioinformatic pathway analysis for multiple miRNA effect indicates that these exercise-responsive miRNAs are involved in the regulation of PI-3-kinase-Akt, focal adhesion, insulin, mTOR and MapK signaling pathways, all of which are modulated in the brain by exercise (Shen et al., 2001; Tong et al., 2001; Bruel-Jungerman et al., 2009; Muller et al., 2011; Elfving et al., 2013) (S6).

Both the sedentary and exercised SAMP8 mice showed altered expression patterns of protein deacetylases with reported functions in the aging brain and AD such as *Hdac6, Sirt1, Hdac3, and Hdac5*. We found a downregulation of histone deacetylase *Hdac6* in the hippocampus of sedentary SAMP8 mice. HDAC6 specific inhibitors have been described as potential therapeutic approaches to rescue the neurodegeneration, however an induction of HDAC6 was reported to facilitate the autophagy of misfolded proteins and aggregates of Aβ 42 and p-tau (Simoes-Pires et al., 2013). Further research is required to better understand the still controversial role of HDAC6 and our data indicate that the SAMP8 mice may represent a suitable model for this purpose. We also found a downregulation of the protein deacetylase *Sirt1* in the hippocampus of SAMP8 mice, supporting the notion that decreased *Sirt1* expression is a feature of the accelerated brain aging and neurodegeneration (Pallas et al., 2008b; Duan, 2013). However, we did not find a modulation of *Sirt1* mRNA levels in response to the running intervention in contrast to previous findings using other experimental models (Ferrara et al., 2008; Dumke et al., 2009; Koltai et al., 2010).

Our data suggest that exercise may exert some of its reported beneficial effects on SAMP8 cognitive performance through *Hdac3* downregulation, a mechanism involved in long-term memory enhancement (Mcquown et al., 2011) and in the reversion of contextual memory deficits in a mouse model of AD (Fischer et al., 2007; Kilgore et al., 2010).

It has previously been reported that exercise activates *Bdnf* transcription through *Hdac5* downregulation (Gomez-Pinilla et al., 2011). Therefore, the downregulation of *Hdac5* expression in the exercised SAMP8 mice may be at least in part responsible for the observed *Bdnf* upregulation (Figure 2). Finally, global acetylation levels of histone H3 (H3ac) were increased after the exercise intervention in the SAMP8 mice suggesting that HDAC gene downregulation had some impact on chromatin remodeling. Notably, partial correlation analyses revealed a negative association between the modification H3ac and *Hdac3* gene expression [ρ_(11)_ = −0.721, *p* < 0.01].

As a whole, our study highlights some common epigenetic features in hippocampus of SAMP8 mice and human AD, and provides further support for the suitability of this experimental model for future epigenetic studies regarding the onset and progression of AD. Among them, miRNAs emerge as potentially valuable biomarkers for the development of new therapeutic strategies for senescence and neurodegeneration. Moreover, our data suggest a positive impact of voluntary exercise in reversing some epigenetic and transcriptional alterations associated with the aging brain, and reinforces the prevailing concept that physical training is a promising therapeutic strategy for neurodegenerative diseases such as AD.

### Conflict of interest statement

The authors declare that the research was conducted in the absence of any commercial or financial relationships that could be construed as a potential conflict of interest.
