# Supplementary material for: Epigenetic alterations in hippocampus of SAMP8 senescent mice and modulation by voluntary physical exercise
Source: Front Aging Neurosci. 2014 Mar 20;6:51. doi: 10.3389/fnagi.2014.00051 (PMC3960508; doi:10.3389/fnagi.2014.00051)
Supplement: Supplementary file 1 [file DataSheet1.PDF]

Supplementary information

S1. List of primers and probe sets used for real time RT-PCR analysis.

Gene Symbol	Reference (ABI)	Reference Sequence ID
Bdnf	Mm01334042_m1	NM_001048142.1
Hdac1	Mm02391771_g1	NM_008228.2
Hdac2	Mm00515108_m1	NM_008229.2
Hdac3	Mm00515916_m1	NM_010411.2
Hdac5	Mm01246076_m1	NM_001077696.1; NM_010412.3
Hdac6	Mm01341125_m1	NM_001130416.1; NM_010413.3
Neuritin	Mm00467844_m1	NM_153529.1
P-300	Mm00625535_m1	NM_177821.6
Sirt1	Mm00490758_m1	NM_001159589.1
Tbp	Mm00446971_m1	NM_013684.3
TrkB	Mm00435422_m1	NM_001025074.1, NM_008745.2

S2. Two-way ANOVA analysis results of hippocampal miRNAs found unaltered in 8-month-old SAMP8 mice compared with SAMR1 mice and unresponsive to the exercise intervention. miRNAs were quantified using the *Mouse Neurological Development & Disease miScript miRNA PCR Array*. *P* values <0.05 were considered statistically significant. Df stands for degrees of freedom.

	**Exercise**			**Strain**			**Strain*exercise**		
	df.	*F*	*p-value*	df.	*F*	*p-value*	df.	*F*	*p-value*
let-7b-5p	1, 12	0.007	0.935	1, 12	1.241	0.287	1, 12	0.086	0.774
let-7c-5p	1, 12	0.213	0.653	1, 12	3.818	0.074	1, 12	2.695	0.127
let-7d-5p	1, 12	0.217	0.650	1, 12	0.475	0.504	1, 12	0.906	0.360
let-7e-5p	1, 12	2.517	0.139	1, 12	1.369	0.265	1, 12	4.235	0.062
miR_9_3p	1, 10	0,117	0,739	1, 10	3,149	0,106	1, 10	1,034	0,333
miR-101a-3p	1, 12	1.113	0.312	1, 12	1.302	0.276	1, 12	0.664	0.431
miR-101b-3p	1, 11	0.440	0.521	1, 11	1.632	0.228	1, 11	0.762	0.401
miR-106b-5p	1, 12	0.012	0.915	1, 12	8.510	*0.013*	1, 12	5.092	*0.043*
miR-107-3p	1, 12	1.161	0.302	1, 12	4.130	0.065	1, 12	0.298	0.595
miR-124-3p	1, 12	1.455	0.251	1, 12	2.219	0.162	1, 12	1.565	0.235
miR-126a-5p	1, 12	5.759	*0.034*	1, 12	3.529	0.085	1, 12	0.475	0.504
miR-130a-3p	1, 12	4.584	0.053	1, 12	1.004	0.336	1, 12	0.153	0.702
miR-132-3p	1, 12	1.533	0.239	1, 12	0.559	0.469	1, 12	0.192	0.669
miR-134-5p	1, 9	0.243	0.634	1, 9	0.826	0.387	1, 9	0.678	0.432
miR-135b-5p	1, 12	0.952	0.349	1, 12	0.740	0.407	1, 12	0.274	0.610
miR-146a-5p	1, 11	0.048	0.830	1, 11	1.421	0.258	1, 11	0.007	0.935
miR-151-3p	1, 12	0.431	0.524	1, 12	5.011	*0.045*	1, 12	0.566	0.466
miR-152-3p	1, 12	1.967	0.186	1, 12	11.180	*0.006*	1, 12	0.446	0.517
miR-15a-5p	1, 12	1.000	0.337	1, 12	0.020	0.891	1, 12	0.441	0.519
miR-181c-5p	1, 12	0.903	0.361	1, 12	3.413	0.089	1, 12	0.659	0.433
miR-181d-5p	1, 12	0.135	0.719	1, 12	3.558	0.084	1, 12	0.535	0.478
miR-191-5p	1, 12	0.861	0.372	1, 12	3.607	0.082	1, 12	0.543	0.475
miR-193b-3p	1, 12	0.303	0.592	1, 12	1.005	0.336	1, 12	0.420	0.529
miR-195a-5p	1, 12	0.421	0.529	1, 12	3.717	0.078	1, 12	0.106	0.75
miR-19b-3p	1, 12	0.195	0.666	1, 12	2.186	0.165	1, 12	2.013	0.181
miR-203-3p	1, 12	0.091	0.768	1, 12	2.144	0.169	1, 12	0.365	0.557
miR-20a-5p	1, 12	0.326	0.578	1, 12	1.008	0.335	1, 12	0.108	0.748
miR-20b-5p	1, 11	1.400	0.262	1, 11	0.023	0.882	1, 11	1.377	0.265
miR-22-3p	1, 12	0.444	0.518	1, 12	1.515	0.242	1, 12	0.111	0.745
miR-24-3p	1, 12	1757	0.210	1, 12	4.517	0.055	1, 12	1.259	0.284
miR-26b-5p	1, 12	0.161	0.696	1, 12	0.076	0.788	1, 12	0.417	0.531
miR-27a-3p	1, 12	1.089	0.317	1, 12	0.008	0.931	1, 12	0.011	0.920
miR-298-5p	1, 12	0.902	0.361	1, 12	0.687	0.423	1, 12	0.424	0.527
miR-29b-3p	1, 12	0.836	0.379	1, 12	0.600	0.453	1, 12	0.756	0.402
miR-30d-5p	1, 12	0.005	0.947	1, 12	4.341	0.059	1, 12	0.086	0.774
miR-320-3p	1, 12	0.911	0.359	1, 12	0.391	0.543	1, 12	0.784	0.393
miR-328-3p	1, 12	0.394	0.542	1, 12	2.901	0.114	1, 12	1.181	0.298
miR-33-5p	1, 12	2.444	0.144	1, 12	0.600	0.453	1, 12	0.629	0.443
miR-338-3p	1, 12	0.407	0.536	1, 12	0.997	0.338	1, 12	0.524	0.483
miR-339-5p	1, 12	2.529	0.138	1, 12	1.778	0.207	1, 12	0.189	0.671
miR-346-5p	1, 12	0.659	0.433	1, 12	2.386	0.148	1, 12	1.115	0.312
miR-34a-5p	1, 12	5.557	*0.036*	1, 12	1.561	0.235	1, 12	0.111	0.745
miR-376b-3p	1, 12	0.002	0.969	1, 12	1.593	0.231	1, 12	1.002	0.337
miR-381-3p	1, 11	0.629	0.445	1, 11	1120	0.313	1, 11	0.437	0.522
miR-409-3p	1, 11	3233	0.100	1, 11	2890	0.117	1, 11	0.681	0.427
miR-433-3p	1, 10	18.826	*0.001*	1, 10	0.423	0.530	1, 10	2.022	0.185
miR-484	1, 12	1.307	0.275	1, 12	1.062	0.323	1, 12	1.058	0.324
miR-485-5p	1, 12	0.303	0.592	1, 12	4.768	0.050	1, 12	3.265	0.096
miR-485-3p	1, 12	1.683	0.219	1, 12	0.047	0.832	1, 12	0.276	0.609
miR-488-3p	1, 12	<0.001	0.991	1, 12	1.212	0.293	1, 12	0.232	0.639
miR-489-3p	1, 12	0.077	0.786	1, 12	2.737	0.124	1, 12	0.334	0.574
miR-598-3p	1, 12	2.951	0.112	1, 12	4.253	0.062	1, 12	1.745	0.211
miR-652-3p	1, 12	0.025	0.878	1, 12	0.005	0.946	1, 12	0.512	0.488
miR-9-5p	1, 12	0.089	0.771	1, 12	1.474	0.248	1, 12	0.205	0.659
miR-92a-3p	1, 11	0.568	0.467	1, 11	0.025	0.876	1, 11	0.003	0.957
miR-93-5p	1, 12	<0.001	0.998	1, 12	0.257	0.621	1, 12	0.362	0.558

*MiR-302a-5p, miR-302b-5p and miR-509-3p were not detected by the real time PCR array.
MiR-106b-5p, miR-126a-5p, miR-151-3p, miR-152-3p, miR-34a-5p and miR-433-3p data were excluded due to the presence of more than one outlier in the same group or due to marginal effects between groups.

S3. Summarized functional information about microRNAs altered in 8-month-old SAMP8 mice.

Mature miRNA ID or Gene Symbol	miRBase or NCBI Accession No.	Reported role/targets in CNS and neurological disorders*	References
**let-7i-(5p)**	MIMAT0000122	Downregulated in the white matter of the superior and middle temporal cortex of AD patients and in the anterior temporal cortex of AD patients.<br>Targets **APP**.	(Hebert et al., 2008;Wang et al., 2011;Nix and Bastiani, 2013)
**mmu-miR-125b-(5p)**	MIMAT0000136	Implicated in cell proliferation, differentiation and migration in neural stem/progenitor cells by targeting **Nestin**.<br>Targets **NR2A** (NMDA receptor subunit) mRNA in hippocampal neurons from mouse brain.<br>Upregulated in AD hippocampus, cerebellum and cortex.<br>Implicated in neurotrophic support, defense against reactive oxygen and nitrogen species and neuroprotection in the CNS.	(Lukiw, 2007;Lukiw and Pogue, 2007;Cogswell et al., 2008;Maes et al., 2009;Edbauer et al., 2010;Boissart et al., 2012;Cui et al., 2012;Zhao et al., 2013)
**mmu-miR-128-(3p)**	MIMAT0000140	Specific from neurons (cultures of embryonic neurons, mouse brian).<br>Involved in neuronal differentiation and neurogenesis.<br>Upregulated in AD hippocampus.<br>Targets **Nicastrin (NCSTN)**, one component of the preseniline complex (γ-secretase activity).	(Lukiw, 2007;Evangelisti et al., 2009;Maes et al., 2009;Bruno et al., 2011;Mallick and Ghosh, 2011)
**mmu-miR-138-(5p)**	MIMAT0000150	Downregulated in the gray matter of cerebral cortex, in hippocampus and prefrontal cortex of AD patients. Associated with long-lasting forms of memory and involved in the control of dendritic spine morphogenesis.	(Siegel et al., 2009;Wang et al., 2011;Lau et al., 2013)
**mmu-miR-140-(5p)**	MIMAT0000151	Targets **EGR2** (transcription factor), modulating myelination in dorsal root ganglion and Schwann cell co-cultures.<br>Downregulated in white matter from superior and middle temporal gyri from AD patients.	(Viader et al., 2011;Im and Kenny, 2012;Majer et al., 2012;Wang et al., 2012)
**mmu-miR-146b-(5p)**	MIMAT0000158	Downregulated in hippocampus, cortex, cerebellum and CSF of AD patients.<br>Involved in innate immune response, induced by NF-kappaB.	(Cogswell et al., 2008;Braidy et al., 2011)

S3 (continuation)

**mmu-miR-181a-(3p)**<br><br><br><br><br>**mmu-miR-181a-(5p)**	MIMAT0000660<br><br><br><br><br><br>MIMAT0000210	Regulated by dopamine.<br>Involved in controlling **GLUA2** (AMPA receptor) surface expression in hippocampal neurons.<br>Dysregulated in postmortem prefrontal cortex from schizophrenia patients.<br>Downregulated in the white matter of the superior and middle temporal cortex of AD patients.	(Perkins et al., 2007;Saba et al., 2008;Beveridge et al., 2010;Wang et al., 2011)
**mmu-miR-342-(3p)**	MIMAT0000590	Downregulated in the gray matter of the superior and middle temporal cortex of AD patients.<br>Upregulated in lymphoblastoid cell lines derived from Autism Spectrum Disorder patients.<br>Regulated by MeCP2 in Rett syndrome.	(Saba et al., 2008;Sarachana et al., 2010;Urdinguio et al., 2010;Wang et al., 2011)

Abbreviations: AD: Alzheimer Disease; **Aβ**: beta-amyloid; **miRNA**: microRNA; **Rb**: Retinoblastoma protein; **BACE1**: beta-site APP-cleaving enzyme 1; **CSF**: Cerebrospinal fluid.

* The microRNA information was collected from scientific works that reported the 3p/5p specification on the microRNA nomenclature applying the current nomenclature conventions, but also from older articles that did not.

S4. Summarized functional information about microRNAs regulated by 8 weeks of voluntary exercise in SAMP8 and SAMR1 mice.

Mature miRNA ID or Gene Symbol	miRBase or NCBI Accession No.	Reported role/targets in CNS and neurological disorders, and exercise regulation) *	References
**mmu-miR-7a-(5p)**	MIMAT0000677	Dysregulated in postmortem prefrontal cortex from schizophrenia patients (miR-7) and in the brain of a Rett syndrome mice model.<br>Downregulated in the grey matter of the superior and middle temporal cortex of AD patients.<br>Controls dopaminergic neurons phenotype of the adult mouse forebrain.<br>Upregulated in human blood natural killer cells and blood mononuclear cells after an acute-aerobic exercise (running).	(Perkins et al., 2007;Beveridge et al., 2010;Wu et al., 2010;Wang et al., 2011;de Chevigny et al., 2012;Radom-Aizik et al., 2012;Radom-Aizik et al., 2013)
**mmu-miR-28a-(5p)**	MIMAT0000653	Upregulated in the cerebral cortex of a transgenic mouse model of AD.	(Wang et al., 2009)
**mmu-miR-98-(5p)**	MIMAT0000545	Downregulated in cerebellum from AD patients, in gray matter from the superior and middle temporal cortex in female AD patients and in mouse primary hippocampal neurons treated with Aβ.<br>Upregulated in the cerebral cortex of a transgenic mouse model of AD.<br>Lower levels are associated with an alteration of the Rb/E2F pathway.	(Ranganathan et al., 2001;Cogswell et al., 2008;Wang et al., 2009;Schonrock et al., 2010;Wang et al., 2011;Satoh, 2012)
**mmu-miR-105**	MIMAT0004856	Upregulated in the prefrontal cortex of individuals with schizophrenia and schizoaffective disorder.	(Perkins et al., 2007)
**mmu-miR-133b-(3p)**	MIMAT0000769	Involved in midbrain dopamine-secreting neurons development, neurite outgrowth and functional recovery after stroke.<br>Dysregulated in the midbrain of Parkinson disease patients and in lymphoblastoid cell lines derived from autism spectrum disorder patients.<br>Downregulated by aerobic exercise in both human and mouse myocytes.	(Nielsen et al., 2010;Sarachana et al., 2010;Soci et al., 2011;Mouradian, 2012;Xin et al., 2013)
**mmu-miR-148b-(3p)**	MIMAT0000580	Downregulated in lymphoblastoid cell lines derived from autistic spectrum disorder patients.<br>Dysregulated in postmortem prefrontal cortex of schizophrenic patients.<br>Downregulated in white matter from superior and middle temporal gyri from AD patients.	(Sarachana et al., 2010;Im and Kenny, 2012;Wang et al., 2012)

Abbreviations: AD: Alzheimer Disease; **Aβ**: beta-amyloid; **miRNA**: microRNA; **Rb**: Retinoblastoma protein; **BACE1**: beta-site APP-cleaving enzyme 1; **CSF**: Cerebrospinal fluid.
* The microRNA information was collected from scientific works that reported the 3p/5p specification on the microRNA nomenclature applying the current nomenclature conventions, but also from older articles that did not.

S5. Biological pathways for miRNAs significantly altered in sedentary SAMP8 compared with SAMR1 mice. Analysis was performed using DIANA mirPath v 2.1 and KEGG software (Vlachos et al., 2012). False discovery rate (FDR) method, a correction for multiple hypothesis testing, and a p-value threshold of 0.05 was used to perform the enrichment analysis.

Biological pathways associated with miRNAs regulated by aging based on DIANA-mirPath analysis

KEGG pathway	p-value	Number of target genes	miRNAs
Prion diseases	1.51E-14	8	7
PI3K-Akt signaling pathway	1.51E-14	82	10
Focal adhesion	1.51E-14	55	11
Acute myeloid leukemia	1.65E-14	22	8
ErbB signaling pathway	9.27E-14	30	9
mTOR signaling pathway	2.42E-13	24	10
Prostate cancer	3.15E-13	29	10
Dorso-ventral axis formation	5.97E-12	11	7
MAPK signaling pathway	3.16E-11	63	10
Mucin type O-Glycan biosynthesis	3.51E-11	12	8
Pathways in cancer	8.31E-11	73	11
Neurotrophin signaling pathway	1.12E-10	35	10
Chronic myeloid leukemia	2.40E-10	25	10
Non-small cell lung cancer	3.29E-10	21	9
TGF-beta signaling pathway	5.71E-10	27	10
Long-term potentiation	1.37E-09	22	9
Axon guidance	1.37E-09	39	11
Renal cell carcinoma	3.57E-09	25	11
Insulin signaling pathway	4.22E-09	36	11
Lysine degradation	4.26E-09	17	9
Pancreatic cancer	5.77E-09	23	9
Endometrial cancer	7.27E-09	18	8
Fc epsilon RI signaling pathway	4.66E-08	22	8
T cell receptor signaling pathway	5.87E-08	29	10
Glycosphingolipid biosynthesis - lacto and neolacto series	9.57E-08	8	7
Bladder cancer	1.85E-07	15	9
Glioma	6.61E-07	21	9
Chagas disease (American trypanosomiasis)	9.70E-07	27	10
Thyroid cancer	1.46E-06	10	9
Protein processing in endoplasmic reticulum	2.66E-06	40	10
Toxoplasmosis	3.51E-06	27	10
Melanoma	4.92E-06	20	9
Fc gamma R-mediated phagocytosis	4.92E-06	23	10
Melanogenesis	4.92E-06	26	10
Other types of O-glycan biosynthesis	6.69E-06	10	8
Small cell lung cancer	6.69E-06	22	10
Regulation of actin cytoskeleton	7.47E-06	47	9
Ubiquitin mediated proteolysis	8.72E-06	34	11

S5 (continuation)

KEGG pathway	p-value	Number of target genes	miRNAs
Glycosaminoglycan biosynthesis - keratan sulfate	2.19E-05	6	6
Osteoclast differentiation	2.23E-05	28	9
Hypertrophic cardiomyopathy (HCM)	2.45E-05	21	9
Wnt signaling pathway	3.61E-05	37	11
Transcriptional misregulation in cancer	1.19E-04	40	11
Amoebiasis	1.95E-04	25	8
ECM-receptor interaction	3.47E-04	20	8
p53 signaling pathway	4.00E-04	17	8
Sphingolipid metabolism	4.65E-04	14	7
Dopaminergic synapse	4.67E-04	30	9
Hedgehog signaling pathway	5.19E-04	13	7
SNARE interactions in vesicular transport	5.19E-04	10	8
Hepatitis C	6.90E-04	28	9
B cell receptor signaling pathway	7.30E-04	18	9
GnRH signaling pathway	8.11E-04	20	7
Glutamatergic synapse	8.11E-04	26	8
Dilated cardiomyopathy	1.09E-03	20	9
Colorectal cancer	1.46E-03	17	9
Hepatitis B	1.49E-03	33	11
Amyotrophic lateral sclerosis (ALS)	1.82E-03	14	8
HTLV-I infection	2.05E-03	52	11
Pertussis	2.47E-03	17	8
VEGF signaling pathway	2.75E-03	15	10
Apoptosis	2.78E-03	19	8
NOD-like receptor signaling pathway	3.61E-03	15	10
Long-term depression	4.29E-03	15	9
Protein digestion and absorption	5.27E-03	18	9
Steroid biosynthesis	1.03E-02	6	6
Progesterone-mediated oocyte maturation	1.04E-02	18	9
Calcium signaling pathway	1.08E-02	33	9
Alanine, aspartate and glutamate metabolism	1.10E-02	9	4
Cholinergic synapse	1.15E-02	24	9
Notch signaling pathway	1.39E-02	11	9
Phosphatidylinositol signaling system	1.40E-02	17	9
Amphetamine addiction	1.47E-02	17	7
Circadian rhythm	1.47E-02	9	8
Gap junction	1.66E-02	19	9
Chemokine signaling pathway	1.69E-02	34	11
Adherens junction	2.07E-02	20	10
Taurine and hypotaurine metabolism	2.51E-02	3	2
Toll-like receptor signaling pathway	2.54E-02	19	8
Aldosterone-regulated sodium reabsorption	2.62E-02	9	8
mRNA surveillance pathway	2.62E-02	20	9
Influenza A	3.10E-02	30	10

S5 (continuation)

KEGG pathway	p-value	Number of target genes	miRNAs
HIF-1 signaling pathway	3.16E-02	22	9
Salmonella infection	3.16E-02	15	8
Basal cell carcinoma	3.47E-02	12	9
Endocytosis	3.56E-02	39	11
N-Glycan biosynthesis	3.90E-02	11	7
Jak-STAT signaling pathway	4.28E-02	27	11

S6. Biological pathways for miRNAs significantly modulated by exercise. Analysis was performed using DIANA mirPath v 2.1 and KEGG software (Vlachos et al., 2012). False discovery rate (FDR) method, a correction for multiple hypothesis testing, and a p-value threshold of 0.05 was used to perform the enrichment analysis.

Biological pathways associated with miRNAs regulated by exercise based on DIANA-mirPath analysis

KEGG pathway	p-value	Number of target genes	miRNAs
PI3K-Akt signaling pathway	1.73E-15	57	5
ECM-receptor interaction	1.52E-10	17	4
Amoebiasis	2.07E-09	22	5
Regulation of actin cytoskeleton	7.46E-09	35	5
Chronic myeloid leukemia	9.15E-09	17	6
Hedgehog signaling pathway	2.60E-08	13	4
TGF-beta signaling pathway	1.12E-07	17	6
Focal adhesion	2.66E-07	31	6
Melanoma	7.87E-07	15	5
Acute myeloid leukemia	1.50E-06	13	5
Pathways in cancer	3.76E-06	45	6
mTOR signaling pathway	7.53E-06	13	5
Insulin signaling pathway	7.53E-06	22	6
Glycosphingolipid biosynthesis - lacto and neolacto series	1.21E-05	5	4
Adherens junction	1.95E-05	16	5
Pancreatic cancer	5.75E-05	13	5
Basal cell carcinoma	5.75E-05	11	5
HTLV-I infection	5.75E-05	36	6
Endometrial cancer	7.21E-05	11	5
Dilated cardiomyopathy	7.30E-05	15	5
Protein processing in endoplasmic reticulum	7.30E-05	25	6
Melanogenesis	1.02E-04	16	5
Hypertrophic cardiomyopathy (HCM)	1.03E-04	14	5
Prostate cancer	1.15E-04	15	5
MAPK signaling pathway	1.18E-04	33	6
Fc gamma R-mediated phagocytosis	1.34E-04	15	6
Protein digestion and absorption	2.02E-04	14	5
Basal transcription factors	3.58E-04	9	4
Axon guidance	4.28E-04	22	6
ErbB signaling pathway	6.64E-04	14	5
Glioma	6.91E-04	12	5
Salmonella infection	9.56E-04	12	4
Adipocytokine signaling pathway	1.31E-03	11	4
Chagas disease (American trypanosomiasis)	1.35E-03	16	5

S6 (continuation)

KEGG pathway	p-value	Number of target genes	miRNAs
Non-small cell lung cancer	1.52E-03	10	5
Long-term potentiation	1.52E-03	11	6
Dorso-ventral axis formation	3.34E-03	5	4
Bacterial invasion of epithelial cells	5.82E-03	10	4
Small cell lung cancer	6.59E-03	12	5
Salivary secretion	8.69E-03	11	4
Colorectal cancer	1.18E-02	10	5
Regulation of autophagy	2.15E-02	6	4
Chemokine signaling pathway	2.48E-02	21	6
Valine, leucine and isoleucine biosynthesis	2.74E-02	1	1
Calcium signaling pathway	2.74E-02	20	5
Lysine degradation	2.88E-02	7	5
Hepatitis C	2.88E-02	16	5
Transcriptional misregulation in cancer	2.88E-02	21	6
Gastric acid secretion	3.14E-02	10	5
Proximal tubule bicarbonate reclamation	3.29E-02	4	3
Aldosterone-regulated sodium reabsorption	4.41E-02	6	4
GnRH signaling pathway	4.41E-02	11	5
Jak-STAT signaling pathway	4.66E-02	17	5
Vasopressin-regulated water reabsorption	4.66E-02	7	5

References S3 and S4

Beveridge, N.J., Gardiner, E., Carroll, A.P., Tooney, P.A., and Cairns, M.J. (2010). Schizophrenia is associated with an increase in cortical microRNA biogenesis. *Mol Psychiatry* 15, 1176-1189. doi: 10.1038/mp.2009.84.

Boissart, C., Nissan, X., Giraud-Triboult, K., Peschanski, M., and Benchoua, A. (2012). miR-125 potentiates early neural specification of human embryonic stem cells. *Development* 139, 1247-1257. doi: 10.1242/dev.073627.

Braidy, N., Guillemin, G.J., Mansour, H., Chan-Ling, T., Poljak, A., and Grant, R. (2011). Age related changes in NAD+ metabolism oxidative stress and Sirt1 activity in wistar rats. *PLoS One* 6, e19194. doi: 10.1371/journal.pone.0019194.

Bruno, I.G., Karam, R., Huang, L., Bhardwaj, A., Lou, C.H., Shum, E.Y., Song, H.W., Corbett, M.A., Gifford, W.D., Gecz, J., Pfaff, S.L., and Wilkinson, M.F. (2011). Identification of a microRNA that activates gene expression by repressing nonsense-mediated RNA decay. *Mol Cell* 42, 500-510. doi: 10.1016/j.molcel.2011.04.018.

Cogswell, J.P., Ward, J., Taylor, I.A., Waters, M., Shi, Y., Cannon, B., Kelnar, K., Kemppainen, J., Brown, D., Chen, C., Prinjha, R.K., Richardson, J.C., Saunders, A.M., Roses, A.D., and Richards, C.A. (2008). Identification of miRNA changes in Alzheimer's disease brain and CSF yields putative biomarkers and insights into disease pathways. *J Alzheimers Dis* 14, 27-41.

Cui, Y., Xiao, Z., Han, J., Sun, J., Ding, W., Zhao, Y., Chen, B., Li, X., and Dai, J. (2012). MiR-125b orchestrates cell proliferation, differentiation and migration in neural stem/progenitor cells by targeting Nestin. *BMC Neurosci* 13, 116. doi: 10.1186/1471-2202-13-116.

De Chevigny, A., Core, N., Follert, P., Gaudin, M., Barbry, P., Beclin, C., and Cremer, H. (2012). miR-7a regulation of Pax6 controls spatial origin of forebrain dopaminergic neurons. *Nat Neurosci* 15, 1120-1126. doi: 10.1038/nn.3142.

Edbauer, D., Neilson, J.R., Foster, K.A., Wang, C.F., Seeburg, D.P., Batterton, M.N., Tada, T., Dolan, B.M., Sharp, P.A., and Sheng, M. (2010). Regulation of synaptic structure and function by FMRP-associated microRNAs miR-125b and miR-132. *Neuron* 65, 373-384. doi: 10.1016/j.neuron.2010.01.005.

Evangelisti, C., Florian, M.C., Massimi, I., Dominici, C., Giannini, G., Galardi, S., Bue, M.C., Massalini, S., Mcdowell, H.P., Messi, E., Gulino, A., Farace, M.G., and Ciafre, S.A. (2009). MiR-128 up-regulation inhibits Reelin and DCX expression and reduces neuroblastoma cell motility and invasiveness. *FASEB J* 23, 4276-4287. doi: 10.1096/fj.09-134965.

Hebert, S.S., Horre, K., Nicolai, L., Papadopoulou, A.S., Mandemakers, W., Silahtaroglu, A.N., Kauppinen, S., Delacourte, A., and De Strooper, B. (2008). Loss of microRNA cluster miR-29a/b-1 in sporadic Alzheimer's disease correlates with increased BACE1/beta-secretase expression. *Proc Natl Acad Sci U S A* 105, 6415-6420. doi: 10.1073/pnas.0710263105.

Im, H.I., and Kenny, P.J. (2012). MicroRNAs in neuronal function and dysfunction. *Trends Neurosci* 35, 325-334. doi: 10.1016/j.tins.2012.01.004.

Lau, P., Bossers, K., Janky, R., Salta, E., Frigerio, C.S., Barbash, S., Rothman, R., Sierksma, A.S., Thathiah, A., Greenberg, D., Papadopoulou, A.S., Achsel, T., Ayoubi, T., Soreq, H., Verhaagen, J., Swaab, D.F., Aerts, S., and De Strooper, B. (2013). Alteration of the microRNA network during the progression of Alzheimer's disease. *EMBO Mol Med* 5, 1613-1634. doi: 10.1002/emmm.201201974.

Lukiw, W.J. (2007). Micro-RNA speciation in fetal, adult and Alzheimer's disease hippocampus. *Neuroreport* 18, 297-300. doi: 10.1097/WNR.0b013e3280148e8b.

Lukiw, W.J., and Pogue, A.I. (2007). Induction of specific micro RNA (miRNA) species by ROS-generating metal sulfates in primary human brain cells. *J Inorg Biochem* 101, 1265-1269. doi: 10.1016/j.jinorgbio.2007.06.004.

Maes, O.C., Chertkow, H.M., Wang, E., and Schipper, H.M. (2009). MicroRNA: Implications for Alzheimer Disease and other Human CNS Disorders. *Curr Genomics* 10, 154-168. doi: 10.2174/138920209788185252.

Majer, A., Medina, S.J., Niu, Y., Abrenica, B., Manguiat, K.J., Frost, K.L., Philipson, C.S., Sorensen, D.L., and Booth, S.A. (2012). Early mechanisms of pathobiology are revealed by transcriptional temporal dynamics in hippocampal CA1 neurons of prion infected mice. *PLoS Pathog* 8, e1003002. doi: 10.1371/journal.ppat.1003002.

Mallick, B., and Ghosh, Z. (2011). A complex crosstalk between polymorphic microRNA target sites and AD prognosis. *RNA Biol* 8, 665-673. doi: 10.4161/rna.8.4.15584.

Mouradian, M.M. (2012). MicroRNAs in Parkinson's disease. *Neurobiol Dis* 46, 279-284. doi: 10.1016/j.nbd.2011.12.046.

Nielsen, S., Scheele, C., Yfanti, C., Akerstrom, T., Nielsen, A.R., Pedersen, B.K., and Laye, M.J. (2010). Muscle specific microRNAs are regulated by endurance exercise in human skeletal muscle. *J Physiol* 588, 4029-4037. doi: 10.1113/jphysiol.2010.189860.

Nix, P., and Bastiani, M. (2013). Neuroscience. Heterochronic genes turn back the clock in old neurons. *Science* 340, 282-283. doi: 10.1126/science.1237921.

Perkins, D.O., Jeffries, C.D., Jarskog, L.F., Thomson, J.M., Woods, K., Newman, M.A., Parker, J.S., Jin, J., and Hammond, S.M. (2007). microRNA expression in the prefrontal cortex of individuals with schizophrenia and schizoaffective disorder. *Genome Biol* 8, R27. doi: 10.1186/gb-2007-8-2-r27.

Radom-Aizik, S., Zaldivar, F., Haddad, F., and Cooper, D.M. (2013). Impact of brief exercise on peripheral blood NK cell gene and microRNA expression in young adults. *J Appl Physiol* 114, 628-636. doi: 10.1152/japplphysiol.01341.2012.

Radom-Aizik, S., Zaldivar, F., Jr., Leu, S.Y., Adams, G.R., Oliver, S., and Cooper, D.M. (2012). Effects of exercise on microRNA expression in young males peripheral blood mononuclear cells. *Clin Transl Sci* 5, 32-38. doi: 10.1111/j.1752-8062.2011.00384.x.

Ranganathan, S., Scudiere, S., and Bowser, R. (2001). Hyperphosphorylation of the retinoblastoma gene product and altered subcellular distribution of E2F-1 during Alzheimer's disease and amyotrophic lateral sclerosis. *J Alzheimers Dis* 3, 377-385.

Saba, R., Goodman, C.D., Huzarewich, R.L., Robertson, C., and Booth, S.A. (2008). A miRNA signature of prion induced neurodegeneration. *PLoS One* 3, e3652. doi: 10.1371/journal.pone.0003652.

Sarachana, T., Zhou, R., Chen, G., Manji, H.K., and Hu, V.W. (2010). Investigation of post-transcriptional gene regulatory networks associated with autism spectrum disorders by microRNA expression profiling of lymphoblastoid cell lines. *Genome Med* 2, 23. doi: 10.1186/gm144.

Satoh, J. (2012). Molecular network of microRNA targets in Alzheimer's disease brains. *Exp Neurol* 235, 436-446. doi: 10.1016/j.expneurol.2011.09.003.

Schonrock, N., Ke, Y.D., Humphreys, D., Staufenbiel, M., Ittner, L.M., Preiss, T., and Gotz, J. (2010). Neuronal microRNA deregulation in response to Alzheimer's disease amyloid-beta. *PLoS One* 5, e11070. doi: 10.1371/journal.pone.0011070.

Siegel, G., Obernosterer, G., Fiore, R., Oehmen, M., Bicker, S., Christensen, M., Khudayberdiev, S., Leuschner, P.F., Busch, C.J., Kane, C., Hubel, K., Dekker, F., Hedberg, C., Rengarajan, B., Drepper, C., Waldmann, H., Kauppinen, S., Greenberg, M.E., Draguhn, A., Rehmsmeier, M., Martinez, J., and Schratt, G.M. (2009). A functional screen implicates microRNA-138-dependent regulation of the depalmitoylation enzyme APT1 in dendritic spine morphogenesis. *Nat Cell Biol* 11, 705-716. doi: 10.1038/ncb1876.

Soci, U.P., Fernandes, T., Hashimoto, N.Y., Mota, G.F., Amadeu, M.A., Rosa, K.T., Irigoyen, M.C., Phillips, M.I., and Oliveira, E.M. (2011). MicroRNAs 29 are involved in the improvement of ventricular compliance promoted by aerobic exercise training in rats. *Physiol Genomics* 43, 665-673. doi: 10.1152/physiolgenomics.00145.2010.

Urdinguio, R.G., Fernandez, A.F., Lopez-Nieva, P., Rossi, S., Huertas, D., Kulis, M., Liu, C.G., Croce, C.M., Calin, G.A., and Esteller, M. (2010). Disrupted microRNA expression caused by Mecp2 loss in a mouse model of Rett syndrome. *Epigenetics* 5, 656-663. doi: 10.4161/epi.5.7.13055.

Viader, A., Chang, L.W., Fahrner, T., Nagarajan, R., and Milbrandt, J. (2011). MicroRNAs modulate Schwann cell response to nerve injury by reinforcing transcriptional silencing of dedifferentiation-related genes. *J Neurosci* 31, 17358-17369. doi: 10.1523/JNEUROSCI.3931-11.2011.

Vlachos, I.S., Kostoulas, N., Vergoulis, T., Georgakilas, G., Reczko, M., Maragkakis, M., Paraskevopoulou, M.D., Prionidis, K., Dalamagas, T., and Hatzigeorgiou, A.G. (2012). DIANA miRPath v.2.0: investigating the combinatorial effect of microRNAs in pathways. *Nucleic Acids Res* 40, W498-504. doi: 10.1093/nar/gks494.

Wang, W., Kwon, E.J., and Tsai, L.H. (2012). MicroRNAs in learning, memory, and neurological diseases. *Learn Mem* 19, 359-368. doi: 10.1101/lm.026492.112.

Wang, W.X., Huang, Q., Hu, Y., Stromberg, A.J., and Nelson, P.T. (2011). Patterns of microRNA expression in normal and early Alzheimer's disease human temporal cortex: white matter versus gray matter. *Acta Neuropathol* 121, 193-205. doi: 10.1007/s00401-010-0756-0.

Wang, X., Liu, P., Zhu, H., Xu, Y., Ma, C., Dai, X., Huang, L., Liu, Y., Zhang, L., and Qin, C. (2009). miR-34a, a microRNA up-regulated in a double transgenic mouse model of Alzheimer's disease, inhibits bcl2 translation. *Brain Res Bull* 80, 268-273. doi: 10.1016/j.brainresbull.2009.08.006.

Wu, H., Tao, J., Chen, P.J., Shahab, A., Ge, W., Hart, R.P., Ruan, X., Ruan, Y., and Sun, Y.E. (2010). Genome-wide analysis reveals methyl-CpG-binding protein 2-dependent regulation of microRNAs in a mouse model of Rett syndrome. *Proc Natl Acad Sci U S A* 107, 18161-18166. doi: 10.1073/pnas.1005595107.

Xin, H., Li, Y., Liu, Z., Wang, X., Shang, X., Cui, Y., Gang Zhang, Z., and Chopp, M. (2013). Mir-133b Promotes Neural Plasticity and Functional Recovery after Treatment of Stroke with Multipotent Mesenchymal Stromal Cells in Rats Via Transfer of Exosome-Enriched Extracellular Particles. *Stem Cells*. doi: 10.1002/stem.1409.

Zhao, Y., Bhattacharjee, S., Jones, B.M., Hill, J., Dua, P., and Lukiw, W.J. (2013). Regulation of Neurotropic Signaling by the Inducible, NF-kB-Sensitive miRNA-

125b in Alzheimer's Disease (AD) and in Primary Human Neuronal-Glial (HNG) Cells. *Mol Neurobiol*. doi: 10.1007/s12035-013-8595-3.
